# Individual differences in voluntary alcohol intake in rats: relationship with impulsivity, decision making and Pavlovian conditioned approach

**DOI:** 10.1007/s00213-017-4617-6

**Published:** 2017-04-18

**Authors:** Marcia Spoelder, Jacques P. Flores Dourojeanni, Kathy C. G. de Git, Annemarie M. Baars, Heidi M. B. Lesscher, Louk J. M. J. Vanderschuren

**Affiliations:** 1 Division of Behavioural Neuroscience, Department of Animals in Science and Society, Faculty of Veterinary Medicine, Utrecht University, Yalelaan 2, 3584CM Utrecht, The Netherlands; 2 Department of Cognitive Neuroscience, Donders Institute for Brain, Cognition and Behaviour, Radboud University Medical Centre, Nijmegen, The Netherlands; 3 Department of Translational Neuroscience, Brain Center Rudolf Magnus, University Medical Center Utrecht, Utrecht, The Netherlands

**Keywords:** Alcohol, Decision making, Impulsivity, Individual differences, Pavlovian-conditioned approach

## Abstract

**Rationale:**

Alcohol use disorder (AUD) has been associated with suboptimal decision making, exaggerated impulsivity, and aberrant responses to reward-paired cues, but the relationship between AUD and these behaviors is incompletely understood.

**Objectives:**

This study aims to assess decision making, impulsivity, and Pavlovian-conditioned approach in rats that voluntarily consume low (LD) or high (HD) amounts of alcohol.

**Methods:**

LD and HD were tested in the rat gambling task (rGT) or the delayed reward task (DRT). Next, the effect of alcohol (0–1.0 g/kg) was tested in these tasks. Pavlovian-conditioned approach (PCA) was assessed both prior to and after intermittent alcohol access (IAA). Principal component analyses were performed to identify relationships between the most important behavioral parameters.

**Results:**

HD showed more optimal decision making in the rGT. In the DRT, HD transiently showed reduced impulsive choice. In both LD and HD, alcohol treatment increased optimal decision making in the rGT and increased impulsive choice in the DRT. PCA prior to and after IAA was comparable for LD and HD. When PCA was tested after IAA only, HD showed a more sign-tracking behavior. The principal component analyses indicated dimensional relationships between alcohol intake, impulsivity, and sign-tracking behavior in the PCA task after IAA.

**Conclusions:**

HD showed a more efficient performance in the rGT and DRT. Moreover, alcohol consumption enhanced approach behavior to reward-predictive cues, but sign-tracking did not predict the level of alcohol consumption. Taken together, these findings suggest that high levels of voluntary alcohol intake are associated with enhanced cue- and reward-driven behavior.

## Introduction

Alcohol is consumed by many people on a regular basis, but only a minority (3–5%) of the people that consume alcohol develop an alcohol use disorder (AUD) (Anthony et al. 1994; Costanzo et al. 2007; United Nations Office on Drugs and Crime 2012; American Psychiatric Association 2013). It is therefore of great relevance to identify the factors that underlie the individual vulnerability to AUD. Importantly, AUD has been associated with exaggerated levels of impulsivity and suboptimal decision making (Kreek et al. 2005; Perry and Carroll 2008; Redish et al. 2008; de Wit 2009; MacKillop et al. 2011) as well as an approach tendency towards reward-predictive cues (Field et al. 2005; Wiers et al. 2007; Field and Cox 2008).

Impulsive behaviors, i.e., the propensity to act without consideration of possible consequences, can be categorized into impulsive action and impulsive choice (Evenden 1999; Reynolds et al. 2006; Pattij and Vanderschuren 2008; Eagle and Baunez 2010; Dalley et al. 2011; Winstanley 2011; Hamilton et al. 2015; Caswell et al. 2015). Both types of impulsivity, as well as suboptimal decision making, have been associated with the susceptibility for AUD (Bates and Labouvie 1997; Dom et al. 2006; Ernst et al. 2006; Verdejo-Garcia et al. 2008; de Wit 2009; Fernie et al. 2010; Goudriaan et al. 2011; King et al. 2011; Fernie et al. 2013). Conversely, excessive alcohol use has also been shown to result in exaggerated impulsivity and suboptimal decision making (Vuchinich and Simpson 1998; Petry 2001; Field et al. 2007; Perry and Carroll 2008; Salgado et al. 2009; Kim et al. 2011; MacKillop et al. 2011; Tomassini et al. 2012; Voon et al. 2013). Together, these findings suggest a complex bidirectionality between impaired impulse control and decision making on the one hand and AUD on the other. Importantly, acute alcohol treatment in rodents and healthy humans has been reported to have mixed effects, i.e., either impaired or unaffected decision making, impulsive action, and impulsive choice (Evenden and Ryan 1999; Richards et al. 1999; George et al. 2005; Perry and Carroll 2008; MacKillop et al. 2011; Mitchell et al. 2011; Semenova 2012; Caswell et al. 2013; Mejia-Toiber et al. 2014; Peña-Oliver et al. 2014). In this regard, it is important to consider that the effects of alcohol may be different in individuals at risk for AUD, since acute alcohol exposure results in reduced behavioral control in heavy drinkers as well as in alcohol pre-exposed rats (Marczinski et al. 2007; Reed et al. 2012; Spoelder et al. 2015b; Sanchez-Roige et al. 2016).

Drug-associated cues can acquire incentive motivational properties that drive conditioned responses towards substances of abuse (Stewart et al. 1984; O’Brien et al. 1998; Robinson and Berridge 2001; Shaham et al. 2003; Milton and Everitt 2010; Tomie and Sharma 2013). Interestingly, substantial individual variation between animals and humans exists with regard to the behavioral response to reward-predictive cues (Zener 1937; Brown and Jenkins 1968; Wilcove and Miller 1974; Burns and Domjan 1996; Tomie et al. 2000, 2012; Cole and Adamo 2005; Stacy and Wiers 2010; Meyer et al. 2012). That is, some individuals approach and manipulate the cue, the so-called sign-trackers, whereas other individuals approach the location of reward delivery, the so-called goal-trackers. In preclinical studies, the rats that show a tendency to acquire a sign-tracking conditioned response have been characterized as more prone to addictive (Flagel et al. 2007, 2008, 2010; Saunders and Robinson 2010, 2011; Yager and Robinson 2013; Yager et al. 2015) and impulsive behavior (Flagel et al. 2010; Lovic et al. 2011). There are interesting human parallels to these findings, since heavy alcohol-drinking individuals exhibit enhanced approach behavior to alcohol-related pictures (Field et al. 2005; Wiers et al. 2009) and approach behavior towards alcohol cues predicts a higher level of alcohol consumption (Palfai 2006; Fadardi and Cox 2008; Christiansen et al. 2012).

In the present study, we assessed whether individual variability in voluntary alcohol consumption relates to differences in impulsivity, decision making, and Pavlovian-conditioned approach. For this purpose, we exploited the substantial individual differences in alcohol intake (Simms et al. 2008; Momeni and Roman 2014; Lesscher et al. 2015; Spoelder et al. 2015a), which we have previously related to the development of compulsive characteristics of alcohol use (Spoelder et al. 2015a, 2017). Groups of rats that voluntarily consume low (low drinkers (LD)) and high (high drinkers (HD)) quantities of alcohol were compared for decision making in a rat gambling task (rGT) (Zeeb et al. 2009; Spoelder et al. 2015b) and for impulsive choice in a delayed reward task (DRT) (Evenden and Ryan 1996; van Gaalen et al. 2006; Baarendse and Vanderschuren 2012). We hypothesized that the consumption of high amounts of alcohol results in maladaptive decision making and enhanced impulsive choice behavior. In addition, we assessed the effects of acute systemic alcohol challenges on stable choice behavior in the rGT and DRT in these rats. Based on our earlier findings, we hypothesized that treatment with moderate doses of alcohol results in a decrement in the number of omissions and provokes impulsive action as assessed by premature responses in both the rGT and DRT, especially in HD (Spoelder et al. 2015b). Finally, we compared LD and HD for approach behavior towards reward-predictive cues (to assess sign- vs goal tracking behavior), whereby we expected that high alcohol consumption induces a sign-tracking phenotype.

## Materials and methods

### Animals

Two groups (experiment 1: *n* = 64; experiment 2: *n* = 80) of male Lister Hooded rats (Charles River, Germany), weighing 220–250 g (~7–9 weeks old) on arrival were used. The rats were individually housed under controlled temperature and humidity conditions on a reversed 12 h light/dark cycle (lights off 7:00 a.m.) with ad libitum access to water and chow. The rats were acclimatized to the housing conditions for 2 weeks before experiments commenced, and they were weighed and handled at least once per week. The rats were briefly restrained during the weighing procedure, to habituate them to the injection procedure. One week before the start of the experiments in the operant conditioning chambers, the rats were gradually restricted to 4–5 g chow 100 g^−1^ body weight day^−1^, which maintained them at 90% of their free-feeding weight. Two days before the introduction to the operant conditioning chambers, the rats received sucrose pellets (45 mg/pellet, TestDiet, UK) in their home cage to reduce potential food neophobia. The same sucrose pellets were used in all behavioral tasks. Behavioral experiments in the operant conditioning chambers were conducted once per day for 5–6 days week^−1^. All experiments were approved by the Animal Ethics Committee of Utrecht University and conducted in agreement with Dutch laws (Wet op de dierproeven, 1996) and European regulations (Guideline 86/609/EEC).

### Experiments

**Fig. 1:**
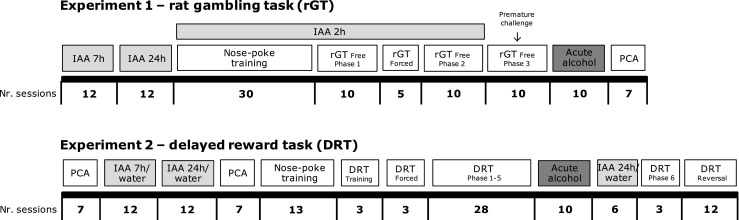
Timeline of the experimental procedures in experiments 1 and 2. *IAA* intermittent alcohol access, *rGT* rat gambling task, *PCA* Pavlovian-conditioned approach, *DRT* delayed reward task

We performed two experiments using two batches of rats (see Fig. 1 for a timeline of the experimental procedures). In the first experiment, the rats were allowed to drink alcohol in their home cage after which low (LD) and high alcohol drinking rats (HD) were selected. Subsequently, these LD and HD were trained and tested in the rGT, received alcohol challenges in the rGT, and were finally tested in the Pavlovian-conditioned approach task. In the second experiment, the rats were first tested for Pavlovian-conditioned approach and were subsequently allowed to drink alcohol in their home cage (*n* = 64), whereby we included a control group that received only water (WAT; *n* = 16). Next, the selected LD and HD and WAT rats were again tested in the Pavlovian-conditioned approach task. Thereafter, these rats were trained and tested in the DRT and received acute alcohol challenges in the DRT. Subsequently, the LD and HD were allowed to drink alcohol in their home cage again and were re-tested on the DRT. Finally, only the LD and HD were tested in a reversed version of the DRT.

### Voluntary home cage alcohol consumption (experiments 1 and 2)

Alcohol access and subgroup selection was performed as previously described (Spoelder et al. 2015a). Briefly, the rats received access to 20% alcohol (*v*/*v* from 99.5%, Klinipath, The Netherlands) and water in a two-bottle choice intermittent alcohol access (IAA) setup in the home cage. The rats received alcohol for 3 days week^−1^, for 7 h day^−1^ in the first month and 24 h day^−1^ in the second month of the experiment. Alcohol intake (g/kg body weight) and preference (percentage of alcohol intake of total fluid intake) were calculated per rat per session and were averaged per week (i.e., 3 sessions week^−1^). In order to select rats that consistently consumed low or high levels of alcohol throughout the experiment, the rats were ranked from low to high based on the rats’ average alcohol intake per week and were assigned ranking scores. These weekly ranking scores were then summed to calculate a total ranking score per rat which was used to divide the rats into subgroups. Rats within the lower and upper 12.5% of the total ranking score range were designated as low and high alcohol drinking rats (LD; HD), respectively. The middle 75% were assigned as medium alcohol-drinking rats; these were used in other experiments. To demonstrate the maintenance of the LD and HD phenotypes at the time of behavioral testing, the rats of experiment 1 were subjected to 2 h IAA after behavioral testing (between 15:00 p.m. and 17:00 p.m.) during nose-poke training and the first 25 rGT sessions (Fig. 1). IAA was ceased after 25 rGT sessions to avoid alcohol consumption to interfere with the acute alcohol challenges that were scheduled after choice behavior in the rGT had stabilized.

### Apparatus (experiments 1 and 2)

Training and testing were conducted in operant conditioning chambers, illuminated by a white house light, in ventilated sound-attenuating cubicles (Med Associates, St. Albans, VT, USA). The Pavlovian-conditioned approach task was conducted in operant chambers with two 4.8-cm-wide retractable levers placed 11.7 cm apart and 6 cm from the grid floor with a magazine between the levers. The chambers used for the rGT and the DRT were equipped with an array of five holes in a curved wall, each with an infrared detector and a stimulus light. In these chambers, a magazine was located on the opposite wall. Sucrose pellets could be delivered via a dispenser in the magazine. Nose pokes into the magazine were detected via an infrared detector. Experimental events and data recording were controlled using MED-PC for Windows.

### Habituation and nose-poke training (experiments 1 and 2)

For the rGT and DRT experiments, a similar habituation and magazine training procedure was used, as described previously (Baarendse and Vanderschuren 2012; Baarendse et al. 2013a; Spoelder et al. 2015b). Briefly, the rats were trained to make a nose-poke response into an illuminated response hole to obtain a sucrose pellet for 30 min or 100 trials/session, whichever occurred first. The rats were trained in three stages during which the stimulus duration was reduced from 30 to 20 and then to 10 s. The inter-trial interval (ITI) was 2 s in the first two training stages and was increased to 5 s in the third and final training stage. The rats progressed to the next training stage after making 30 correct responses in a session. In order to obtain a comparable level of experience in correct performance before the rGT and DRT and to prevent over-training, the rats that quickly approached the performance criterion were tested two to three times per week instead of daily; this occurred in both LD and HD. The training sessions continued until all rats achieved baseline performance, defined by performing ≥80% of the trials correctly for three consecutive days.

### Rat gambling task (experiment 1)

The rGT (Zeeb et al. 2009; Baarendse et al. 2013a, b) was carried out as described previously (Spoelder et al. 2015b). Briefly, the rats could choose from three options (safe, optimal, risky) in which the safe and risky option resulted in a net gain of 72 and 24% of the optimal option, respectively. Only the middle three response holes of the total of five holes in the array were used. The two outer holes were inactive; a nose-poke response into these holes was without programmed consequences. The spatial location of the three options was counterbalanced across subjects; these remained the same for each rat over the course of the experiment. Because we were interested in the capability of LD and HD to optimize their choice behavior over sessions by trial and error (i.e. without prior knowledge of the consequences of each choice), the rats were first tested for ten free choice sessions (phase 1). After inspection of the data of these ten free choice sessions, we noticed that some rats (three HD rats and four LD rats) had not sufficiently explored all three choices (defined as making <20% responses for a given option in any of the ten free choice sessions in phase 1). These rats did not sufficiently sample the safe option (one LD rat, one HD rat), the optimal option (one LD rat, one HD rat), the risky option (one LD rat, one HD rat), or both the optimal and risky option (one LD rat). Therefore, to ensure that all rats had equal experience with the contingencies of the three choice options, the rats were subsequently tested during five forced choice sessions. In phase 2, the rats first received five free choice sessions. Because we observed that several rats had still not explored all three options during these five free choice sessions, the following five free choice sessions were preceded by 10 min of forced choices. In phase 3, the rats were tested for another ten free choice sessions, which resulted in a stable choice pattern.

A trial started with a 5-s ITI, followed by illumination of one (during forced choice sessions) or three (during free choice sessions) stimulus lights for 10 s. A response into an illuminated hole turned off the stimulus light(s) and led to either a reward (i.e., sucrose pellets) or punishment (i.e., no reward delivery and time-out period signaled by a flashing stimulus light within the chosen hole at a frequency of 1 Hz). A nose-poke response into a non-illuminated aperture (i.e., incorrect response), a failure to respond within 10 s (i.e., omission), or a response during the ITI (i.e., premature response), resulted in a 5-s timeout period, signaled by illumination of the house light. Nose-poke responses into the stimulus holes during either punishment or reward were scored as perseverative responses but had no scheduled consequences. The rats were tested for impulsive action by measuring the premature responses during the last ten free choice sessions in the rGT. To provoke impulsive action, the ITI was extended to 7 s in free choice rGT sessions 23 and 28 (Dalley et al. 2007; Baarendse and Vanderschuren 2012).

### Delayed reward task (experiment 2)

A detailed description of the DRT procedure has been provided previously (van Gaalen et al. 2006; Baarendse and Vanderschuren 2012). In short, a trial started with a 5-s ITI after which the middle response hole was illuminated for 10 s. After a response in this hole, the light extinguished and the two response holes adjacent to the middle response hole were illuminated. The DRT session was divided into five blocks of ten trials. Each block started with two forced choice trials in order to signal the upcoming delay for the subsequent session block. During these forced choice trials, either the left or right hole was illuminated in a counterbalanced fashion. For the next eight or ten trials (see below), both the left and right holes were illuminated and the rats could make a choice. Responding in one of the two holes was rewarded with a small reward (one sucrose pellet) provided immediately, whereas responding in the other hole was rewarded with a large reward (four sucrose pellets) after a certain delay. A response into an illuminated hole turned off the stimulus light(s). An incorrect response, an omission, or a premature response resulted in a 5-s timeout period, signaled by the illumination of the house light. Nose-poke responses into the stimulus holes after making a choice were scored as perseverative responses, but these had no scheduled consequences. The delays for the large reward were presented in blocks in an ascending order within a session. The spatial location of the two choices was counterbalanced across subjects and remained the same for each rat over the course of the experiment. As the trial time was fixed, the ITI duration depended on the duration of the delay.

The delays for the large reward were gradually increased over sessions, to ensure that the rats acquired the contingencies of the task. First, the rats were subjected to three sessions with delays for the large reward of 0, 2, 4, 8, and 12 s (phase 1), followed by two sessions with delays of 0, 4, 8, 16, and 24 s (phase 2), one session with delays of 0, 8, 16, 32, and 48 s (phase 3), and six sessions with the final delays of 0, 10, 20, 40, and 60 s (phase 4). In phase 5, the number of choices was extended from eight free choices per delay to the final ten free choices per delay, in which the rats were tested for 16 sessions. As we noted during the first session that not all rats finished all trials when ten free choices were used, we used eight free choice trials/block during phases 1–4 to make sure that the rats finished the session. As training progressed, all animals came to finish all trials, and the number of choices was increased to ten free choice trials, consistent with our previous studies (van Gaalen et al. 2006; Baarendse and Vanderschuren 2012). Subsequently, the rats were exposed to acute alcohol challenges and six 24 h IAA sessions, after which the rats were again tested on the DRT for three sessions (phase 6). It has been suggested that increased choice for the large delayed reward in the DRT can be the result of perseverative responding for the large reward option, rather than a genuine reduction in impulsive choice (Maguire et al. 2014; Orsini et al. 2017). Therefore, to test whether differences between HD and LD rats in the DRT were related to perseverative responding, in phase 7, the delay for the large reward was reversed within the session from 60 to 40, 20, 10, and 0 s. The rats were tested under these conditions for 13 sessions (Fig. 1).

### Pavlovian-conditioned approach task (experiments 1 and 2)

The rats were habituated to the chambers for two sessions, during which 50 sucrose pellets were randomly delivered over the course of 25 min with an average inter-reward interval of 30 s. The Pavlovian-conditioned approach procedure was conducted as previously described (Flagel et al. 2011; Spoelder et al. 2015c). Briefly, a trial consisted of the insertion of the left or right lever (counterbalanced between rats) for 8 s (conditioned stimulus (CS)), followed by the response-independent delivery of a sucrose pellet (unconditioned stimulus (US)). Cue lights above the lever or within the magazine were not illuminated. The rats were subjected to 25 CS–US presentations in each session, which occurred under a variable inter-trial interval schedule, with on average 90 s between trials. Lever contacts and food magazine entries during lever presentation were recorded but had no programmed consequences.

### Systemic alcohol injections (experiments 1 and 2)

Alcohol (99.5%, Klinipath, The Netherlands) was diluted with saline to a concentration of 10% alcohol (*v*/*v*). Injection volumes were adjusted to the body weight and the alcohol dose. Alcohol solutions were prepared fresh daily and administered intraperitoneally 15 min prior to behavioral testing. The syringes were pre-heated to 32 °C by a heating pad to prevent possible decreases in body temperature after injection of substantial volumes, particularly at the highest alcohol doses. Vehicle (i.e., saline) injection volumes were equivalent to the volume required for an injection of the 0.6-g kg^−1^ alcohol dose. Prior to injections, the rats were habituated twice to the injection procedure. The different alcohol challenge doses were administered according to a within-subject, Latin square design with a 3-day cycle for each dose, i.e., a baseline session, followed by an alcohol treatment session and a washout day during which the rats remained in their home cage.

### Data analysis

The behavioral measures to assess task performance in the rGT and DRT were calculated as the percentage choice for a certain option, i.e., (number of choices for a certain option/total number of choices × 100). For the DRT, the area under the curve (AUC) for the overall percentage choice for the large delayed reward was also calculated (Myerson et al. 2001). For the allocation of behavioral responses during the Pavlovian-conditioned approach task, we analyzed the number of lever presses and magazine entries during CS presentations and the response bias score. The response bias score was calculated as ((lever presses − magazine entries)/(lever presses + magazine entries)), resulting in a number ranging from −1 (goal-tracking) to +1 (sign-tracking) (Meyer et al. 2012; Spoelder et al. 2015c). The increase in premature responses when a longer ITI was used in the rGT was calculated as a ratio, i.e., (number of premature responses during long ITI session/the average number of premature responses of the 2 sessions preceding and the 2 sessions following the long ITI session). When data were not normally distributed, data was square root transformed for count data and LOG transformed for latency data, which resulted in the normal distribution of the data in all cases. Thus, prior to statistical analyses, the number of lever presses and head entries during the Pavlovian-conditioned approach task and the number of omissions, premature and perseverative responses during the rGT and DRT were square root transformed and the trial initiation, choice, and collect latencies were LOG transformed. Choice behavior in the rGT, expressed as percentages, was arcsine transformed prior to analysis. The data obtained in the Pavlovian-conditioned approach task, choice behavior in the rGT and the AUC in the DRT were analyzed using one-, two-, and three-way repeated-measure ANOVAs with choice, session, and alcohol treatment as within-subject variables and group (LD, HD, WAT) as the between-subject variable. Mauchly’s test of sphericity was used to test if variances of the differences between treatment levels were equal. If the assumption of sphericity was violated, degrees of freedom were corrected using Huynh-Feldt estimates of sphericity to more conservative values. Corrected degrees of freedom are presented rounded to the nearest integer. Because the nose-poke training prior to the rGT and DRT was performed in a similar manner, these data were analyzed together via univariate ANOVAs with group (LD, HD, WAT) and experiment number (1 and 2) as factors. Since in a small minority of sessions some rats did not respond during a certain delay block, data of the DRT were analyzed with linear mixed models (Verbeke and Molenberghs 2000) in which the delay and group served as variables. By using the linear mixed models, the available data points of the rats with a missing data point could be included in the analyses. The data obtained in the rGT and DRT after alcohol challenges were also analyzed using linear mixed models since we noticed that the rats were less sedated upon treatment with the second high dose compared with the first one (0.8 or 1.0 g kg^−1^). Therefore, we included the injection order, together with dose, delay, and subgroup as variables in the mixed model analyses. For all mixed model analyses, the covariance structure was explored and modeled appropriately. In addition, when significant main effects or interactions with group were detected in the mixed models analyses, *post hoc* pairwise comparisons with a Sidak correction were made. Student’s samples and paired *t* tests were used for post hoc analyses for the comparison of different doses per group. Wilcoxon signed-rank tests were used for post hoc analyses for the comparison of different doses for the percentage of choice for the large delayed reward in the DRT.

The relationships between the behavioral variables under study were further investigated by principal component analyses. The principal component analyses involving the decision-making performance were performed separately for experiments 1 and 2 because different animals were involved. Using Bartlett’s test of sphericity, we verified whether there were relationships between the variables included in the analyses. The variables included from experiment 1 were based on the significant group differences between LD and HD, i.e., (1) the averaged alcohol intake during the 4 weeks of 24 h IAA (24 h IAA), (2) number of sessions to acquire the nose-poke response during training (nose-poke acquisition), (3) the percentage of correct responses during baseline performance (nose-poke performance), (4) the averaged percentage of optimal choices during the final phase of the rGT (optimal choice rGT), (5) the ratio of premature responses during the first long ITI challenge in the last phase of the rGT (impulsive action), and (6) the averaged response bias score (an index of sign- vs. goal-tracking, see above) of the final two sessions of the Pavlovian-conditioned approach task (response bias after IAA). The variables included from experiment 2 were based on the significant group differences between HD, LD, and/or WAT, i.e., (1) 24 h IAA, (2) nose-poke acquisition, (3) nose-poke performance, (4) the averaged percentage choice for the large delayed reward during the fourth phase of the DRT (impulsive choice phase 4), (5) the averaged response bias score of the final two sessions of the Pavlovian-conditioned approach task before IAA/water access (response bias before IAA), and (6) response bias after IAA.

All statistical analyses were conducted using IBM SPSS Statistics for Windows, version 22.0 (IBM Corp., Armonk, NY, USA). The threshold for statistical significance was set at *p* < 0.05. All data are presented as mean ± SEM. Graphs were made using Graphpad Prism 6.

## Results

### Alcohol consumption during IAA in HD and LD (experiments 1 and 2)

**Fig. 2:**
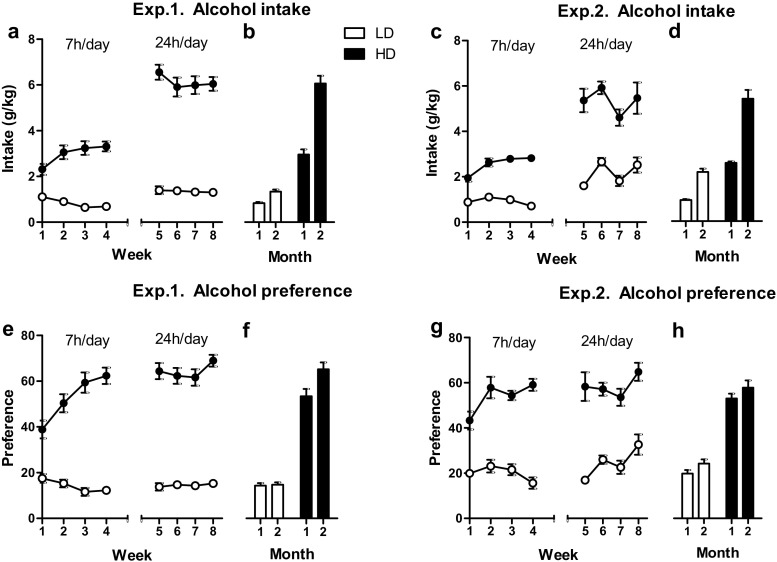
Alcohol consumption (**a**-**d**) and preference (**e**-**h**) in LD and HD during IAA. Alcohol intake and preference (expressed as % alcohol intake of total fluid intake) increased in HD during the first 4 weeks of IAA with 7 h access day^−1^ but remained low in LD. Alcohol intake and preference remained stable during 24 h IAA. HD showed a larger extent of increased alcohol intake than LD when comparing the averaged alcohol intake of the 4 weeks with 7 h access day^−1^ with the averaged alcohol intake of the 4 weeks with 24 h access day^−1^ (**a**-**b**, **c**-**d**). Data are shown as mean ± SEM

Alcohol intake and preference increased over the first 4 weeks of IAA for 7 h day^−1^ in HD but remained stable in LD (intake: exp. 1: *F*_(3, 42) week × group_ = 15.67, *p* < 0.001; exp. 2: *F*_(3, 42) week × group_ = 8.64, *p* < 0.001; preference: exp. 1: *F*_(3, 42) week × group_ = 17.40, *p* < 0.001; exp. 2: *F*_(3, 39) week × group_ = 4.66, *p* < 0.01) (Fig. 2). Upon extension of alcohol access duration to 24 h day^−1^ in the second month of the experiment, alcohol intake increased to a larger extent in HD than in LD (exp. 1: *F*_(1, 14) month × group_ = 78.31, *p* < 0.001; exp. 2: *F*_(1, 14) month × group_ = 12.52, *p* < 0.005) (Fig. 2b, d). The preference for alcohol in experiment 1 increased from the first to the second month in HD but not in LD (*F*_(1, 14) month × group_ = 11.89, *p* < 0.005) (Fig. 2f), whereas the preference for alcohol in experiment 2 increased to a comparable extent in HD and LD (*F*_(1, 14) month × group_ = 0.01, n.s.) (Fig. 2h). Alcohol intake and preference during the 4 weeks of 24 h day^−1^ access remained stable in both groups (intake: exp. 1: *F*_(3, 42) week × group_ = 1.74, n.s.; exp. 2: *F*_(2, 33) week × group_ = 1.13, n.s.; preference: exp. 1: *F*_(3, 42) week × group_ = 2.20, n.s.; exp. 2: *F*_(3, 42) week × group_ = 1.32, n.s.) (Fig. 2). The total fluid intake (i.e., intake of the water and alcohol solutions added up) during the 2 months was not different between the HD and LD in experiment 1, nor between the HD, LD, and WAT in experiment 2 (exp. 1: *F*_(1, 14) group_ = 0.01, n.s.; exp. 2: *F*_(2, 29) group_ = 0.21, n.s.) (data not shown). Importantly, the HD maintained higher levels of alcohol consumption during the 2-h IAA sessions that were incorporated between the nose-poke training and the rGT tests in experiment 1 (intake: LD, 0.77 ± 0.08 g/kg; HD, 1.48 ± 0.06 g/kg; *F*_(1, 14) group_ = 51.17, *p* < 0.001; preference: LD, 39.67 ± 2.73%; HD, 70.14 ± 1.82%; *F*_(1, 14) group_ = 86.06, *p* < 0.001) (data not shown).

### Nose-poke training (experiments 1 and 2)

The number of sessions required to acquire the nose-poke response was dependent on group (LD, HD, WAT) and experiment (exp. 1 or 2) (*F*_(1, 43) experiment_ = 58.78, *p* < 0.001; *F*_(1, 43) experiment × group_ = 4.73, *p* < 0.04; *F*_(2, 43) group_ = 1.58, n.s.) (Table 1). Subsequent analyses of the groups per experiment indicated that the HD and LD in experiments 1 and 2 required a similar number of sessions to acquire the nose-poke response (*t*_(14)_ = 1.53, n.s.; *t*_(14)_ = −1.02, n.s., respectively), whereas the WAT group required less sessions than the LD (*t*_(9)_ = 2.88, *p* < 0.05), but a comparable number of sessions as HD in experiment 2 (*t*_(22)_ = 1.47, n.s.). During baseline performance, the percentage of correct responses was different between the groups (*F*_(2, 43) group_ = 4.86, *p* < 0.02) but not between the experiments (*F*_(1, 43) experiment_ = 2.32, n.s.; *F*_(1, 43) experiment × group_ = 0.21, n.s.). Subsequent analyses of the groups showed a higher percentage of correct responses in HD compared to LD (*t*_(30)_ = 3.09, *p* < 0.005), whereas the water group did not differ from either the HD or LD (*t*_(30)_ = 1.35, n.s.; *t*_(30)_ = −1.57, n.s., respectively) (Table 1). During baseline performance, we observed no differences between the groups with regard to the total number of responses, omissions, premature responses, and the choice latency (*F*_(1, 43) experiment × group_ = 0.01, n.s.; *F*_(2, 43) group_ = 2.03, n.s.; *F*_(1, 43) experiment × group_ = 0.24, n.s.; *F*_(2, 43) group_ = 1.52, n.s.; *F*_(1, 43) experiment × group_ = 0.93, n.s.; *F*_(2, 43) group_ = 0.28, n.s.; *F*_(1, 43) experiment × group_ = 0.01, n.s.; *F*_(2, 43) group_ = 1.25, n.s., respectively). However, we observed that the rats in experiment 2 made a larger number of responses and premature responses, a smaller number of omissions, and had a shorter choice latency (*F*_(1, 43) experiment_ = 8.86, *p* < 0.006; *F*_(1, 43) experiment_ = 21.55, *p* < 0.001; *F*_(1, 43) experiment_ = 9.70, *p* < 0.004; *F*_(1, 43) experiment_ = 8.26, *p* < 0.007, respectively) (Table 1). The reward collection latency was not different between groups or experiments (*F*_(2, 43) group_ = 1.19, n.s.; *F*_(1, 43) experiment_ = 0.00, n.s). Although there was a significant interaction (*F*_(1, 43) experiment × group_ = 4.46, *p* < 0.05), subsequent analyses did not reveal any significant differences.

**Table 1:** Nose-poke training results

	Experiment 1		Experiment 2		
	HD	LD	HD	LD	WAT
Nr sessions to acquire nose-poke response	14.88 ± 2.22	11.13 ± 1.04	4.13 ± 0.64	5.13 ± 0.74	3.25 ± 0.28^#^
Percentage correct during baseline	96.62 ± 0.80^*^	93.50 ± 0.84	95.17 ± 0.71*	91.84 ± 1.56	94.43 ± 0.89
Total responses during baseline	78.71 ± 4.23	77.19 ± 2.44	87.25 ± 1.87	85.21 ± 3.82	91.54 ± 1.15
Omissions during baseline	21.29 ± 4.23	22.81 ± 2.44	12.75 ± 1.87	14.00 ± 3.85	8.46 ± 1.15
Premature during baseline	4.46 ± 1.21	2.73 ± 0.63	10.67 ± 2.25	11.83 ± 2.50	12.40 ± 1.58
Premature of last session in stage 2 (ITI 2 s)	0.50 ± 0.27	1.38 ± 0.84	8.63 ± 2.43	12.38 ± 3.45	11.38 ± 2.39
Premature of first session in stage 3 (ITI 5 s)	10.00 ± 2.90	4.75 ± 1.36	49.75 ± 10.00	39.75 ± 11.51	64.75 ± 8.76
Choice latency (s)	3.08 ± 0.16	3.48 ± 0.11	2.46 ± 0.25	2.85 ± 0.30	2.66 ± 0.16
Collect latency (s)	3.09 ± 0.23	2.48 ± 0.24	2.47 ± 0.19	3.44 ± 0.73	2.32 ± 0.09

Data are shown as the mean ± SEM

^**#**^ *p* < 0.05, significant difference between WAT and LD (post hoc Student’s *t* test); ^*^ *p* < 0.05, significant difference between HD and LD (post hoc Student’s *t* test)

#### Impulsive action

Repeated-measures ANOVA of the number of premature responses in the final session in training stage 2 with an ITI of 2 s and the first session of training stage 3 with an ITI of 5 s, showed an increase in the number of premature responses (*F*_(1, 43) session_ = 116.39, *p* < 0.001) that was dependent on both the group (*F*_(2, 43) session × group_ = 4.01, *p* < 0.03; *F*_(2, 43) group_ = 1.16, n.s.) and the experiment (*F*_(1, 43) session × experiment_ = 5.07, *p* < 0.04; *F*_(1, 43) experiment_ = 33.72, *p* < 0.001; *F*_(1, 43) session × group × experiment_ = 0.02, n.s.). Subsequent analyses per group indicated a trend towards a greater increment in premature responses in the HD compared with the LD in experiment 1 (*F*_(1, 14) session × group_ = 3.30, *p* = 0.091; *F*_(1, 14) group_ = 0.59, n.s.) and in experiment 2 (*F*_(1, 29) session × group_ = 2.58, *p* = 0.093; *F*_(2, 29) group_ = 0.80, n.s.) (Table 1).

### Rat gambling task (experiment 1)

**Fig. 3:**
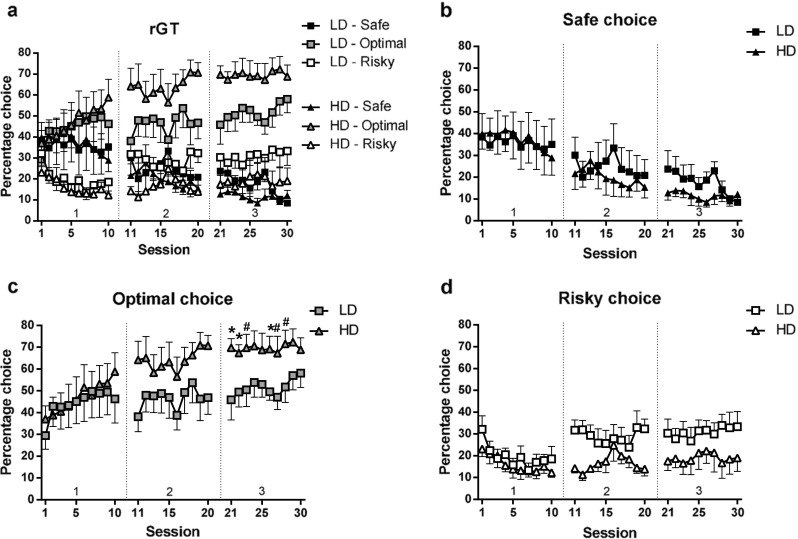
Choice behavior of LD and HD during the different phases of the rGT. Both groups developed a preference for the optimal choice over sessions (**a**). During the ten free choice sessions in phase 3, HD showed a higher percentage choice for the optimal option (**c**) whereas no group differences were observed in the percentage choice for the safe (**b**) and risky option (**d**). Data are shown as the mean percentage choice + SEM. *p < 0.05, significant difference between groups (post hoc Student’s *t* test); ^#^ *p* < 0.07, trend towards a significant difference between groups (post hoc Student’s *t* test). *Numbers 1, 2, and 3 above the x-axis* represent the different phases of the experiment

During the first ten free choice sessions in phase 1 of the rGT, the rats developed a preference for the optimal option (*F*_(18, 252) choice × session_ = 3.18, *p* < 0.001), independent of group (*F*_(18, 252) choice × session × group_ = 0.58, n.s.; *F*_(1, 14) group_ = 0.06, n.s.) (Fig. 3a). Separate analyses per choice indicated that the percentage choice for the safe option did not change (*F*_(9, 126) session_ = 1.50, n.s.), but the percentage choice for the optimal option increased (*F*_(7, 92) session_ = 3.93, *p* < 0.002) and the percentage choice for the risky option decreased over sessions (*F*_(9, 126) session_ = 3.86, *p* < 0.002) (Fig. 3a–d). In the subsequent ten free choice sessions in phase 2, the difference in percentage choice between the three options became significant (*F*_(2, 22) choice_ = 9.97, *p* < 0.003), whereby a similar choice pattern was observed in both groups (*F*_(2, 22) choice × group_ = 1.82, n.s.; *F*_(1, 14) group_ = 0.00, n.s.) (Fig. 3a). In the final phase, the difference in percentage choice between the three options remained (*F*_(2, 28) choice_ = 30.02, *p* < 0.001), and this was different between HD and LD (*F*_(2, 28) choice × group_ = 3.37, *p* < 0.05; *F*_(1, 14) group_ = 5.63, *p* < 0.04) (Fig. 3a). Separate analyses per choice indicated that HD showed a higher percentage choice for the optimal option than LD (*F*_(1, 14) group_ = 4.92, *p* < 0.05) (Fig. 3c). However, the groups did not differ in their percentage choice for the safe (*F*_(1, 14) group_ = 1.56, n.s.) (Fig. 3b) and risky options (*F*_(1, 14) group_ = 2.85, n.s.) (Fig. 3d).

#### Impulsive action

**Fig. 4:**
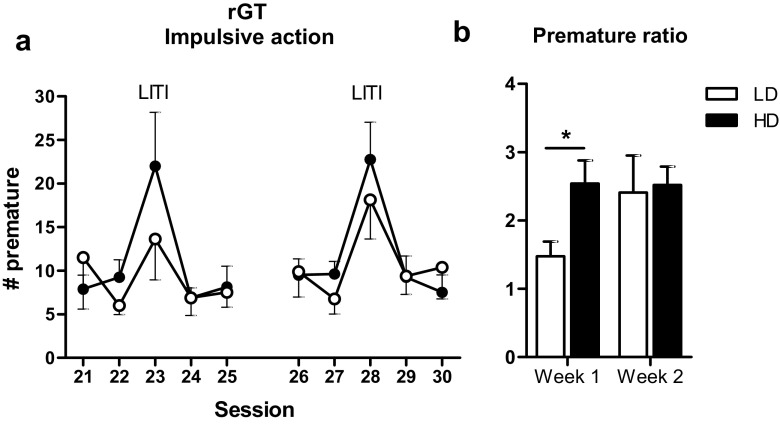
Effects of a long inter-trial interval (*LITI*) in rGT free choice sessions 23 and 28; **a** the number of premature responses (i.e., impulsive action) during the last phase of the rGT. The LITI provoked an increase in premature responding in both groups (**a**). The increase in premature responding was enhanced in HD compared with LD during the first but not during the second LITI session (**a**, **b**). **b** The increase in premature responses during the LITI sessions as a ratio. Data are shown as the mean + SEM. *p < 0.05, significant difference between groups (one-way ANOVA)

During the last ten free choice sessions, a longer ITI (7 s) was used in sessions 23 and 28, which provoked an increase in premature responses in both groups (*F*_(9, 126) session_ = 7.52, *p* < 0.001; *F*_(1, 14) group_ = 0.33, n.s.; *F*_(9 126) group × session_ = 0.87, n.s.) (Fig. 4a). We observed a larger increase in premature responses, expressed as a ratio, in HD compared with LD during the first (*F*_(1, 14) group_ = 7.01, *p* < 0.02) but not during the second long ITI session (*F*_(1, 14) group_ = 0.03, n.s.) (Fig. 4b).

#### Acute alcohol challenges

**Fig. 5:**
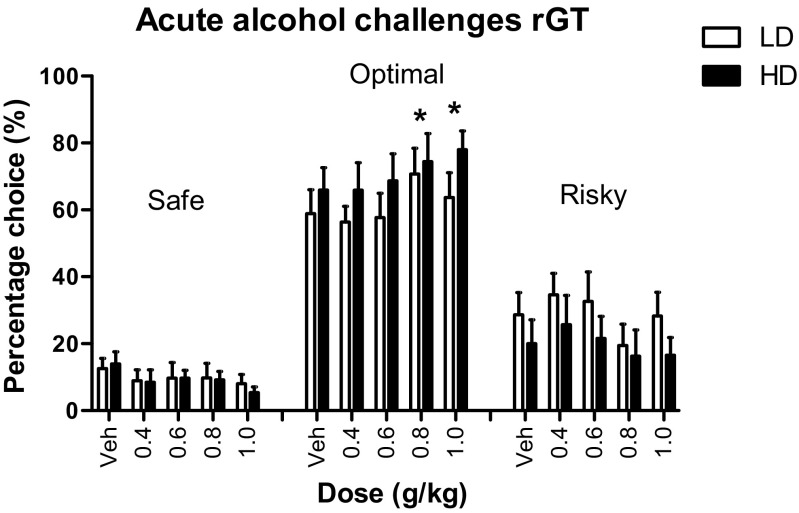
Effects of acute alcohol treatment on choice behavior in the rGT. Alcohol affected choice behavior in LD and HD to a similar extent. Alcohol increased choice for the optimal option but not the safe and risky options. Data are shown as the mean percentage choice + SEM. *p < 0.05, significantly different from vehicle (post hoc paired *t* tests)

Acute alcohol treatment affected choice behavior in the rGT (*F*_(8, 39) dose × choice_ = 5.31, *p* < 0.001), in a comparable manner in LD and HD (*F*_(8, 39) dose × choice × group_ = 1.01, n.s.) (Fig. 5). Subsequent analyses per choice indicated that alcohol treatment dose-dependently increased the percentage choice for the optimal option (*F*_(4, 78) dose_ = 4.09, *p* < 0.006) which was significant after treatment with 0.8 and 1.0 g/kg alcohol. The percentage choice for the safe (*F*_(4, 83) dose_ = 2.14, n.s.) and risky option (*F*_(4, 31) dose_ = 2.15, n.s.) was not affected by acute alcohol treatment (Fig. 5). Treatment with alcohol reduced the number of choices, premature and perseverative responses and increased the number of omissions, choice latencies and collect latencies, whereby these effects were more pronounced in HD (Table 2).

**Table 2:** Effects of alcohol treatment on behavior in the rGT

Variable	Dose effect		Vehicle	0.4 g/kg	0.6 g/kg	0.8 g/kg	1.0 g/kg
Choices	*F*_(4, 64) dose_ = 38.65, *p* < 0.001; *F*_(1, 56) group_ = 3.06, *p* = 0.086; *F*_(4, 64) dose × group_ = 7.91, *p* < 0.001	HD	72.25 ± 7.75	69.38 ± 6.06	62.88 ± 8.23	39.75^#^* ± 4.25	35.13* ± 5.80
		LD	70.75 ± 4.86	71.63 ± 2.93	57.38 ± 4.20	62.13 ± 5.63	45.25* ± 7.01
Omissions	*F*_(4, 64) dose_ = 112.35, *p* < 0.001; *F*_(1, 24) group_ = 1.43, n.s.; *F*_(4, 64) dose × group_ = 14.36, *p* < 0.001	HD	25.13 ± 5.81	18.13 ± 4.71	21.00 ± 7.54	50.88* ± 10.08	69.75* ± 6.81
		LD	14.13 ± 4.58	8.75 ± 2.42	16.13 ± 5.38	29.25 ± 6.60	44.13* ± 8.77
Premature	*F*_(4, 64) dose_ = 35.50, *p* < 0.001; *F*_(1, 8) group_ = 0.70, n.s.; *F*_(4, 64) dose × group_ = 9.32, *p* < 0.001	HD	14.25 ± 4.88	14.00 ± 2.69	10.88 ± 4.15	4.00* ± 1.79	5.38* ± 1.63
		LD	9.00 ± 2.20	7.00 ± 1.55	6.38 ± 1.34	6.50 ± 1.52	4.25* ± 1.03
Perseverative	*F*_(4, 64) dose_ = 48.75, *p* < 0.001; *F*_(1, 8) group_ = 1.06, n.s.; *F*_(4, 64) dose × group_ = 4.25, *p* < 0.005	HD	24.63 ± 5.54	31.00 ± 3.54	22.25 ± 5.98	6.63^#^* ± 2.22	8.63* ± 1.74
		LD	36.75 ± 8.41	40.38 ± 7.68	22.75 ± 3.61	13.00* ± 2.49	18.00* ± 4.80
Choice latency (s)	*F*_(4, 17) dose_ = 59.83, *p* < 0.001; *F*_(1, 17) group_ = 1.49, n.s.; *F*_(4, 17) dose × group_ = 16.10, *p* < 0.001	HD	3.27 ± 0.48	3.13 ± 0.37	3.69* ± 0.50	4.25* ± 0.28	4.28* ± 0.11
		LD	3.07 ± 0.36	3.20 ± 0.34	3.93 ± 0.37	4.01* ± 0.21	3.79* ± 0.20
Collect latency (s)	*F*_(4, 64) dose_ = 11.07, *p* < 0.001; *F*_(1, 45) group_ = 0.01, n.s.; *F*_(4, 64) dose × group_ = 5.97, *p* < 0.001	HD	2.10 ± 0.32	2.15 ± 0.39	2.23 ± 0.19	2.94* ± 0.32	3.72* ± 0.95
		LD	2.63 ± 0.56	2.45 ± 0.42	2.52 ± 0.78	2.53 ± 0.38	2.65 ± 0.41

Data are shown as the mean ± SEM

*p < 0.05, significantly different from vehicle (post hoc paired *t* test); ^#^ *p* < 0.05, significant difference between HD and LD (post hoc student’s *t* test)

### Delayed reward task (experiment 2)

**Fig. 6:**
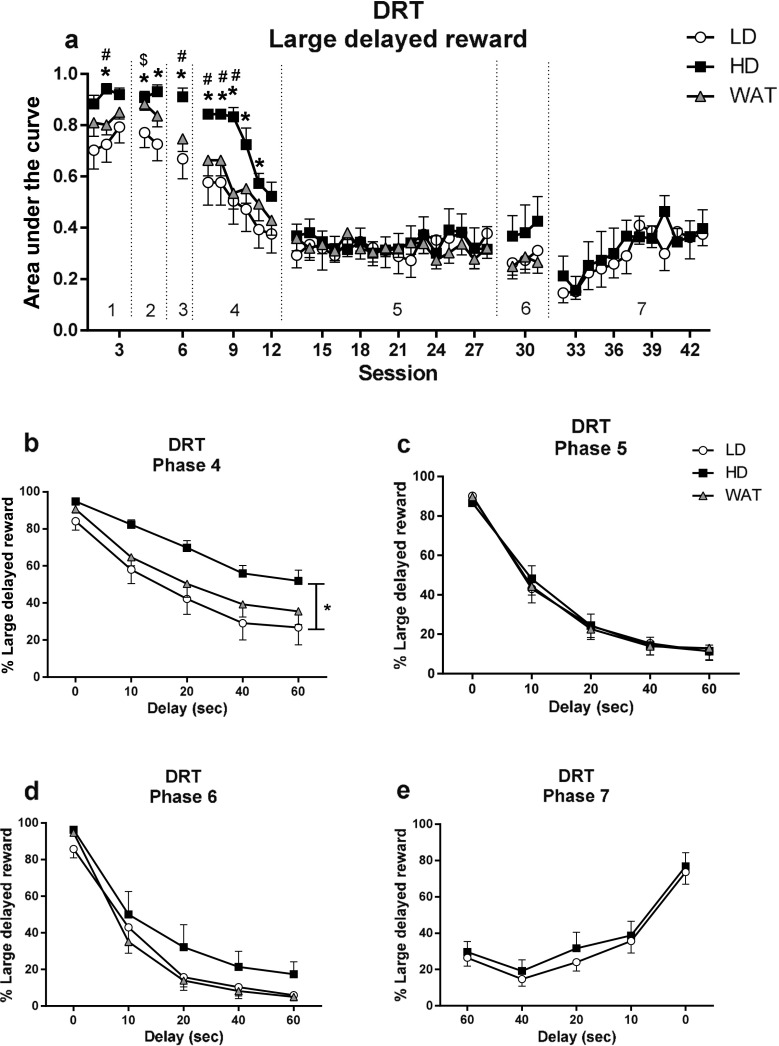
Choice behavior of LD, HD, and WAT during the different phases of the DRT. The area under the curve declined over sessions as the delays to the large reward increased during phases 1–5 (**a**). The preference for the large delayed reward was higher in HD during phases 1–4 (**a**, **b**), but group differences were no longer significant in phase 5 (**c**). Upon re-exposure to alcohol, the group differences re-emerged (**a**, **d**). Reversal of the delays (phase 7) did not differentially affect choice behavior in LD and HD (**a**, **e**). **b**–**e** The averaged choice behavior across all sessions in the phases. Data are shown as the mean percentage choice + SEM. *p < 0.05, significant difference between LD and HD (one-way ANOVA or post hoc Student’s *t* test); ^#^ *p* < 0.05, significant difference between HD and WAT (one-way ANOVA or post hoc Student’s *t* test); and ^$^ *p* < 0.05, significant difference between LD and WAT (one-way ANOVA or post hoc Student’s *t* test)

The AUC in the DRT declined over sessions as the delays increased during training phase 1–5 (*F*_(27, 783) session_ = 58.88, *p* < 0.001), but in a different manner for LD, HD, and WAT (*F*_(54, 783) session × group_ = 1.46, *p* < 0.02; *F*_(2, 29) group_ = 1.77, n.s.) (Fig. 6a). Post hoc analyses per session indicated that HD showed significantly higher AUC values compared with LD and WAT during phases 1–4 (Fig. 6a). Analyses of choice behavior over delays in the different phases of the experiment confirmed group differences for the second and fourth phases (phase 2: *F*_(2, 37) group_ = 3.87, *p* < 0.04; phase 4: *F*_(2, 32) group_ = 3.61, *p* < 0.04; Fig. 6b) and a trend for the first and the third phase (phase 1: *F*_(2, 33) group_ = 2.81, *p* = 0.075; phase 3: *F*_(2, 31) group_ = 3.25, *p* = 0.053). Subsequent post hoc analyses of the second and fourth phases indicated that the HD showed a higher percentage choice for the large delayed reward compared with LD (*p* < 0.04 and *p* < 0.05, respectively), with no differences between HD and WAT or LD with WAT. These group differences were independent of the delays (phase 1: *F*_(4, 44) delay_ = 6.37, *p* < 0.001; *F*_(8, 44) delay × group_ = 1.08, n.s.; phase 2: *F*_(4, 65) delay_ = 3.75, *p* < 0.009; *F*_(8, 65) delay × group_ = 0.71, n.s.; phase 3: *F*_(4, 62) delay_ = 3.35, *p* < 0.02; *F*_(8, 63) delay × group_ = 0.80, n.s.; phase 4: *F*_(4, 66) delay_ = 52.79, *p* < 0.001; *F*_(8, 66) delay × group_ = 0.72, n.s.) (Fig. 6b). There were no group differences during phase 5 (*F*_(2, 32) group_ = 0.00, n.s.; *F*_(4, 49) delay_ = 248.51, *p* < 0.001; *F*_(8, 49) delay × group_ = 0.58, n.s.) (Fig. 6c).

To investigate whether the group differences during phases 1–4 were the residual result of IAA, the LD and HD were re-exposed to six IAA sessions (whereby WAT received water) and were re-tested in the DRT (phase 6). During these IAA sessions, HD consumed more alcohol than LD (*F*_(1, 14) group_ = 30.36, *p* < 0.001) and showed a greater preference for alcohol (*F*_(1, 14) group_ = 45.07, *p* < 0.001). The averaged alcohol intake and preference levels during these six IAA sessions (intake: LD, 1.69 ± 0.34 g kg^−1^ session^−1^; HD, 5.20 ± 0.53 g kg^−1^ session^−1^; preference: LD, 24.31 ± 4.31%; HD, 68.86 ± 5.10%) did not differ from the averaged alcohol intake and preference levels during the 12 24 h IAA sessions in the second month of IAA (intake: LD: 2.15 ± 0.14 g/kg/session, HD: 5.35 ± 0.38 g/kg/session; preference: LD: 24.50 ± 1.84%, HD: 58.47 ± 3.23%) (intake: *F*_(1, 14) time_ = 2.05, n.s.; *F*_(1, 14) time × group_ = 0.58, n.s.; *F*_(1, 14) group_ = 46.10, *p* < 0.001; preference: *F*_(1, 14) time_ = 4.00, n.s.; *F*_(1, 14) time × group_ = 4.46, n.s.; *F*_(1, 14) group_ = 68.24, *p* < 0.001). Comparison of phase 5 and 6 revealed an interaction between group and phase (*F*_(2, 63) phase × group_ = 9.80, *p* < 0.001) indicating that recent IAA differentially affected choice behavior in the three groups of animals (Fig. 6c, d). Post hoc analyses per group revealed that the percentage choice for the large delayed reward was reduced in phase 6 compared with phase 5 in LD and WAT but not in HD (LD: *F*_(1, 58) phase_ = 5.99, *p* < 0.02; HD: *F*_(1,9) phase_ = 2.93, n.s.; WAT: *F*_(1, 86) phase_ = 14.20, *p* < 0.001) (Fig. 6c, d). Separate analyses of phase 6, however, did not reveal a significant group effect (*F*_(2, 32) group_ = 1.73, n.s.; *F*_(4, 50) delay_ = 363.87, *p* < 0.001; *F*_(8, 51) delay × group_ = 1.33, n.s.).

Upon reversal of the delays during the session (phase 7), when only the LD and HD were tested, the overall percentage choice (i.e., AUC) for the large delayed reward progressively increased over sessions, towards baseline performance (*F*_(7, 100) session_ = 6.83, *p* < 0.001; *F*_(7, 100) session × group_ = 0.83, n.s.) (Fig. 6a). Both HD and LD showed a reversal of choice behavior in a delay-dependent manner, without a group difference (*F*_(1, 16) group_ = 0.31, n.s.; *F*_(4, 32) delay_ = 94.12, *p* < 0.001; *F*_(4, 32) delay × group_ = 0.35, n.s.) (Fig. 6e).

#### Acute alcohol challenges

**Fig. 7:**
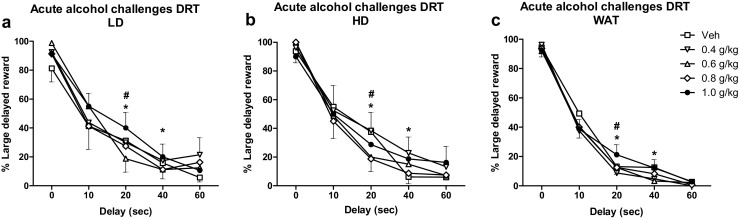
Effects of acute alcohol treatment on choice behavior in the DRT. Alcohol affected choice behavior, depending on the delay to the large reward but independent of group. Alcohol reduced the preference for the large delayed reward alcohol during the 20- and 40-s delay. Data are shown as the mean percentage choice + SEM. For reasons of clarity, the results of the LD, HD, and WAT are shown separately and the SEMs are depicted only for the lowest and highest values in the graph. *p < 0.05, significantly different vehicle at 0.4 g kg^−1^ alcohol (post hoc paired *t* tests); ^#^ *p* < 0.065, significantly different from vehicle at 0.6 g kg^−1^ alcohol (post hoc paired *t* tests)

Acute alcohol treatment affected choice behavior in the DRT in a dose-dependent manner (*F*_(4, 205) dose_ = 4.08, *p* < 0.004), dependent upon delay (*F*_(16, 698) dose × delay_ = 2.54, *p* < 0.002) but independent of group (*F*_(32, 698) dose × delay × group_ = 1.26, n.s.) (Fig. 7). Subsequent analyses per delay (with the WAT, LD and HD groups combined) indicated that, although trends were observed, alcohol treatment did not significantly affect choice behavior during the 0-s delay block (*F*_(4, 47) dose_ = 2.19, *p* = 0.085), the 10-s delay block (*F*_(4, 82) dose_ = 2.24, *p* = 0.071), or the 60-s delay block (*F*_(4, 26) dose_ = 1.37, n.s.). Acute alcohol treatment decreased the percentage choice for the large delayed reward during the 20- and 40-s delay blocks (*F*_(4, 57) dose_ = 3.05, *p* < 0.03; *F*_(4, 49) dose_ = 3.05, *p* < 0.03, respectively). Post hoc analyses indicated that the percentage choice for the large delayed reward during the 20-s delay period decreased after treatment with 0.4 and 0.6 g kg^−1^ alcohol (*p* < 0.04) and the percentage choice for the large delayed reward during the 40-s delay period decreased after treatment with 0.4 g kg^−1^ alcohol (*p* < 0.01) (Fig. 7).

The alcohol challenges reduced the number of trials initiated, the trial initiation latency and the number of perseverative responses, and increased the number of omissions, the choice latency, and the reward collection latency. Alcohol treatment had a comparable effect on the LD, HD, and WAT, except for choice latency, in which the HD and WAT showed an increase in the choice latency at 0.6, 0.8, and 1.0 g kg^−1^, whereas the LD showed an increase in the choice latency at 0.8 and 1.0 g kg^−1^. Group differences were found in the number of omissions, premature responses, and the trial initiation latency, in which LD made more omissions compared with WAT, HD made less premature responses compared with WAT, and LD had longer trial initiation latencies compared with WAT (Table 3).

**Table 3:** Effects of alcohol treatment on behavior in the DRT

Variable	Dose effect		Vehicle	0.4 g/kg	0.6 g/kg	0.8 g/kg	1.0 g/kg
Initiated trials	*F*_(4, 87) dose_ = 8.59, *p* < 0.001; *F*_(2, 33) group_ = 0.74, n.s.; *F*_(8, 87) dose × group_ = 0.57, n.s.	HD	64.75 ± 0.67	66.00 ± 1.05	63.13 ± 2.22	60.25* ± 3.53	57.00* ± 4.13
		LD	65.25 ± 0.65	65.75 ± 1.31	66.13 ± 1.95	63.88* ± 2.58	61.88* ± 3.57
		WAT	65.88 ± 1.47	66.50 ± 1.79	62.75 ± 1.92	63.63* ± 1.19	61.75* ± 1.65
Omissions	*F*_(4, 39) dose_ = 10.47, *p* < 0.001; *F*_(2, 36) group_ = 3.57, *p* < 0.04^a^; *F*_(8, 39) dose × group_ = 0.49, n.s.	HD	21.63 ± 9.19	15.88 ± 4.60	38.50* ± 14.18	43.88* ± 15.13	64.00* ± 21.14
		LD	31.00 ± 6.75	29.88 ± 7.14	36.88* ± 8.71	54.63* ± 12.08	54.25* ± 15.48
		WAT	17.13 ± 7.34	19.69 ± 8.07	26.56* ± 9.18	29.13* ± 9.22	41.63* ± 11.40
Premature	*F*_(4, 160) dose_ = 1.57, n.s.; *F*_(2, 160) group_ = 4.64, *p* < 0.02^b^; *F*_(8, 160) dose × group_ = 0.84, n.s.	HD	5.25 ± 1.83	4.63 ± 1.80	3.38 ± 1.25	2.50 ± 0.94	3.38 ± 1.08
		LD	6.63 ± 2.35	6.25 ± 1.33	4.50 ± 1.25	5.38 ± 1.65	4.00 ± 1.56
		WAT	4.75 ± 0.94	6.63 ± 1.48	5.19 ± 0.78	4.69 ± 0.90	4.06 ± 0.81
Perseverative	*F*_(4, 50) dose_ = 5.32, *p* < 0.002; *F*_(2, 32) group_ = 3.36, *p* < 0.05^c^; *F*_(8, 50) dose × group_ = 2.19, *p* < 0.05	HD	22.38 ± 5.35	31.13 ± 4.45	23.25 ± 5.46	16.63 ± 6.20	15.38 ± 3.20
		LD	24.13 ± 6.32	22.63 ± 5.08	20.75 ± 6.17	26.50 ± 7.88	19.63 ± 4.24
		WAT	26.50 ± 3.93	32.06 ± 3.75	24.56 ± 3.28	27.25 ± 3.91	29.13 ± 5.72
Trial initiate latency (s)	*F*_(4, 128) dose_ = 3.70, *p* < 0.01; *F*_(2, 24) group_ = 3.63, *p* < 0.05^d^; *F*_(8, 128) dose × group_ = 1.46, n.s.	HD	3.29 ± 0.39	2.96* ± 0.33	3.29 ± 0.37	3.53 ± 0.25	3.45 ± 0.41
		LD	3.88 ± 0.18	3.57* ± 0.18	3.74 ± 0.15	3.72 ± 0.12	3.77 ± 0.18
		WAT	3.25 ± 0.13	3.14* ± 0.19	3.18 ± 0.17	3.11 ± 0.16	3.47 ± 0.14
Choice latency (s)	*F*_(4, 38) dose_ = 37.46, *p* < 0.001; *F*_(2, 32) group_ = 2.12, n.s.; *F*_(8, 38) dose × group_ = 2.94, *p* < 0.02	HD	0.43 ± 0.06	0.45 ± 0.05	0.56* ± 0.07	0.51* ± 0.06	0.55* ± 0.08
		LD	0.38 ± 0.03	0.47 ± 0.08	0.48 ± 0.04	0.65* ± 0.09	0.56* ± 0.06
		WAT	0.35 ± 0.02	0.39 ± 0.03	0.44* ± 0.04	0.47* ± 0.05	0.50* ± 0.03
Collect latency (s)	*F*_(4, 46) dose_ = 5.04, *p* < 0.003; *F*_(2, 29) group_ = 2.04, n.s.; *F*_(8, 46) dose × group_ = 1.60, n.s.	HD	2.86 ± 0.48	2.59 ± 0.45	4.08 ± 1.25	3.08* ± 0.36	3.38* ± 0.66
		LD	4.09 ± 1.33	2.87 ± 0.32	3.22 ± 0.30	3.96* ± 0.62	3.24* ± 0.50
		WAT	2.30 ± 0.16	2.80 ± 0.59	5.43 ± 2.86	2.80* ± 0.33	3.81* ± 0.70

Data are shown as the mean ± SEM

*p < 0.05, significantly different from vehicle (post hoc paired *t* tests)

^a^LD made more omissions compared with WAT (*p* < 0.04)

^b^HD made less premature responses compared with WAT (*p* < 0.01)

^c^No sign. Post hoc group differences

^d^LD had longer latencies compared with WAT (*p* < 0.05)

### Pavlovian-conditioned approach (experiments 1 and 2)

**Fig. 8:**
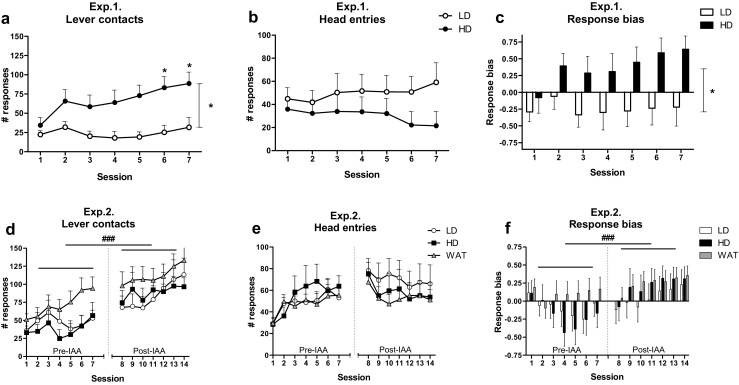
Pavlovian-conditioned approach behavior in LD, HD, and WAT in experiments 1 and 2. In experiment 1 (i.e., after IAA), a higher number of lever contacts was observed in HD compared with LD (**a**), while no differences in the number of head entries during CS presentation were observed (**b**). Moreover, HD showed a higher response bias, reflecting more sign-tracking, compared with LD (**c**). In experiment 2, the number of lever contacts and head entries during CS presentation developed in a different manner prior to and after IAA (whereby WAT had access to water only), but this was independent of group (**d**, **e**). Interestingly, the response bias tended to develop differently between groups before and after IAA (**f**). While no effect of session was apparent prior to IAA, after IAA, a clear increase in response bias, reflecting more sign-tracking was observed, without significant differences between groups. *p < 0.05, significant main effect of group or significant difference between groups within a session (post hoc Student’s *t* test, *p* < 0.03); ^###^ *p* < 0.002, significant main effect of IAA

In the rats in experiment 1, the number of lever contacts increased over sessions (*F*_(4, 50) session_ = 3.76, *p* < 0.02), whereas the head entries into the food magazine during CS presentation remained unchanged (*F*_(5, 73) session_ = 0.73, n.s.) (Fig. 8a, b). The total number of lever contacts was higher in HD than in LD (*F*_(1, 14) group_ = 8.47, *p* < 0.02), and this increased to a further extent over sessions (*F*_(4, 50) session × group_ = 3.01, *p* < 0.04) (Fig. 8a). Post hoc analyses indicated that the number of lever contacts was higher in HD during sessions 6 and 7. The number of head entries into the food magazine during CS presentation was not different between groups (*F*_(1, 14) group_ = 1.73, n.s.; *F*_(5, 73) session × group_ = 1.46, n.s.) (Fig. 8b). As a result, the response bias was higher in HD than in LD (*F*_(1, 14) group_ = 4.98, *p* < 0.05), but this did not develop differently in HD and LD (*F*_(4, 53) session_ = 2.53, *p* = 0.055; *F*_(4, 53) session × group_ = 1.84, n.s.) (Fig. 8c).

To assess whether this difference in approach behavior between HD and LD was a cause or a consequence of the different amounts of alcohol consumed by HD and LD, we assessed Pavlovian-conditioned approach behavior prior to and after IAA (whereby the WAT had access to water only) in experiment 2 (Fig. 1). We observed that the number of lever contacts (*F*_(1, 29) group_ = 41.92, *p* < 0.001) increased in a comparable manner for the three groups of rats (*F*_(2, 29) phase × group_ = 0.96, n.s.) (Fig. 8d). When the development over sessions was analyzed, we found that the number of lever contacts increased in a comparable manner before and after IAA (*F*_(3, 93) phase × session_ = 2.35, *p* = 0.074), independent of group (*F*_(6, 93) phase × session × group_ = 1.72, n.s.) (Fig. 8d). The head entries into the food magazine during CS presentation developed in a different manner before and after IAA (*F*_(4, 125) phase × session_ = 12.92, *p* < 0.001), independent of group (*F*_(9, 125) phase × session × group_ = 0.98, n.s.) (Fig. 8e). Separate analyses indicated an increase in the number of head entries into the food magazine during CS presentation before IAA (*F*_(3, 92) session_ = 4.91, *p* < 0.004), independent of group (*F*_(2, 29) group_ = 0.26, n.s.; *F*_(6, 92) session × group_ = 1.07, n.s.), whereas a decrease was observed after IAA (*F*_(3, 89) session_ = 5.90, *p* < 0.002), independent of group (*F*_(2, 29) group_ = 0.46, n.s.; *F*_(6, 89) session × group_ = 0.58, n.s.) (Fig. 8e). IAA influenced the response bias (*F*_(1, 29) phase_ = 14.16, *p* < 0.002) in a similar manner in the three groups (*F*_(2, 29) phase × group_ = 1.95, n.s.) (Fig. 8f). The response bias followed a different pattern over sessions before and after IAA (*F*_(3, 98) phase × session_ = 9.19, *p* < 0.001) and a trend towards an interaction with the group was found (*F*_(7, 98) phase × session × group_ = 1.87, *p* = 0.085) (Fig. 8f). Separate analyses before IAA indicated no effect of session on the response bias (*F*_(3, 84) session_ = 2.10, n.s.; *F*_(2, 29) group_ = 0.88, n.s.; *F*_(6, 84) session × group_ = 1.77, n.s.). However, after IAA, a clear increase in the response bias over sessions was apparent (*F*_(4, 107) session_ = 13.14, *p* < 0.001), independent of group (*F*_(2, 29) group_ = 0.50, n.s.; *F*_(7, 107) session × group_ = 0.94, n.s.) (Fig. 8f).

### Principal component analyses

**Fig. 9:**
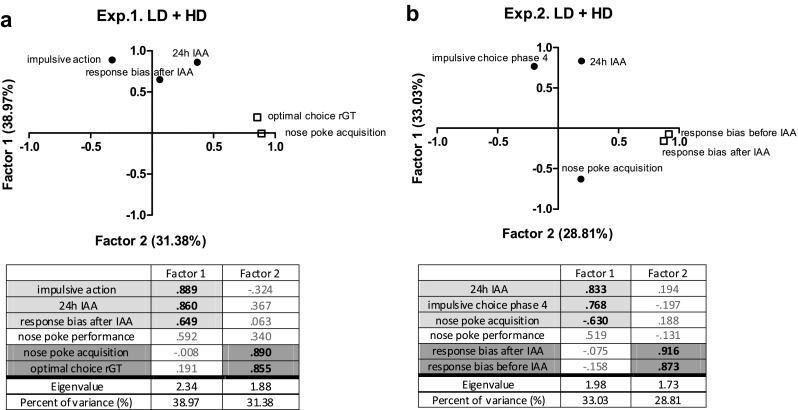
**a** Principal component analysis of the most important variables in experiment 1 revealed two factors explaining 70.35% of the total variance of the model. **b** Principal component analysis of the most important variables in experiment 2 revealed two factors explaining 61.84% of the total variance of the model. Only variables with a loading above 60% were presented in the graphs. *24 h IAA* the averaged alcohol intake during the 4 weeks of 24 h IAA, *nose-poke acquisition* number of sessions to acquire the nose-poke response during training, *nose-poke performance* the percentage of correct responses during baseline performance; *optimal choice rGT* the averaged percentage of optimal choices during the final phase of the rGT, *impulsive action* the ratio of premature responses during the first long ITI challenge in the last phase of the rGT, *response bias after IAA* the averaged response bias score of the final two sessions of the Pavlovian-conditioned approach task, *response bias before IAA* the averaged response bias score of the final two sessions of the Pavlovian-conditioned approach task before IAA, *impulsive choice phase 4* the averaged percentage choice for the large delayed reward during the fourth phase of the DRT

Principal component analyses of both the LD and HD together were performed to determine the relationships between the different behavioral parameters described in the above sections. A principal component analysis of the first experiment revealed two factors explaining 70.35% of the total variance of the model. The first factor, which accounts for 38.97% of the model, includes impulsive action, 24 h IAA, and the response bias after IAA. The second factor, which accounts for 31.38% of the model and is orthogonal to the first one, includes the nose-poke acquisition and the optimal choice rGT (Fig. 9a). A principal component analysis of the second experiment revealed two factors explaining 61.84% of the total variance of the model. The first factor, which accounts for 33.03% of the model, includes 24 h IAA, impulsive choice during phase 4 and the nose-poke acquisition. The second factor, which accounts for 28.81% of the model and is orthogonal to the first one, includes the response bias after IAA and the response bias before IAA (Fig. 9b).

## Discussion

In the present study, we investigated the relationship between cognitive control, cue-directed behavior, and alcohol intake in rats. To that aim, we selected subgroups of HD and LD and assessed choice behavior in the rGT and the DRT, as well as Pavlovian-conditioned approach. The data showed that HD displayed more optimal choice behavior in the rGT, and a transient reduction in impulsive choice in the DRT. Moreover, HD showed more sign-tracking behavior compared with LD, although this was not likely to be a pre-existing trait. Acute alcohol exposure increased the preference for the optimal choice in the rGT and increased impulsive choice in the DRT, but this occurred independent of the level of alcohol intake. The principal component analyses indicated dimensional relationships between alcohol intake, impulsive action, and sign-tracking behavior in the Pavlovian-conditioned approach task after IAA, as well as a relationship between alcohol intake and impulsive choice. These findings shed light on the association between alcohol use, decision making, impulsivity and the behavioral responses to reward-associated cues, suggesting that a high alcohol consumption phenotype is related to enhanced reward- or cue-driven behavior.

We observed a higher percentage of choices for the optimal option in HD in the rGT, when behavior in the task had become stable and well-established. Impairments in decision making and exaggerated levels of impulsivity have generally been observed in AUD patients, and to a lesser extent in binge drinkers and heavy drinkers (Vuchinich and Simpson 1998; Bechara et al. 2001; Petry 2001; Fein et al. 2004; Field et al. 2007; Johnson et al. 2008; Loeber et al. 2009; Salgado et al. 2009; Claus et al. 2011; Gullo and Stieger 2011; MacKillop et al. 2011; Reed et al. 2012; Le Berre et al. 2014; Bickel et al. 2014; Banca et al. 2016). Because HD display key characteristics of AUD, i.e., increased motivation for alcohol and loss of control over alcohol use (Spoelder et al. 2015a, 2017), we hypothesized that HD would show suboptimal decision making. Our findings contrast with this hypothesis. However, one needs to bear in mind that cognitive deficits of this kind are not invariably observed in AUD. For example, no difference or even less risky decision making has been observed in AUD patients in the Balloon Analogue Risk Task (Ashenhurst et al. 2011; Claus and Hutchison 2012). Likewise, several preclinical studies have shown that alcohol exposure during adulthood does not affect decision making or even increases cognitive performance in certain tasks (DePoy et al. 2013; Mejia-Toiber et al. 2014; Schindler et al. 2014). It is important to note that (except for during phase 3) the rats had access to alcohol during rGT training and testing. We do not think, however, that alcohol access or early withdrawal has had an influence of rGT performance. Although the difference in alcohol intake between LD and HD rats remained, the animals ingested relatively modest amounts of alcohol during the 2-h alcohol drinking sessions during rGT training. Moreover, the animals had access to alcohol *after* the daily rGT sessions, making it unlikely that presence of alcohol in the animals’ system influenced behavior. Moreover, cessation of intake of this level of alcohol intake is known not to cause marked withdrawal (e.g., Goldstein 1972). Interestingly, the results of the principal component analyses showed that alcohol intake and rGT performance did not load onto the same factor. Rather, the level of alcohol intake of the LD and HD in the first experiment loaded onto the same factor as impulsive action and the response bias in the Pavlovian-conditioned approach task. Interestingly, enhanced impulsive action, measured as premature responding, has been observed in AUD patients (Voon et al. 2013) and binge drinkers (Sanchez-Roige et al. 2014a, b). In the rGT, we observed a transient increase in the number of premature responses in HD compared with LD during challenge sessions with a long ITI, whereas the groups did not differ in impulsive action during baseline sessions with a 5-s ITI. In line with these findings, HD tended to show a larger increase in impulsive action during nose-poke training when the ITI was increased from 2 to 5 s. It has been reported that group differences in impulsive action can be unmasked or exaggerated by testing the animals under unexpected and challenging task conditions, such as increasing the ITI (Dalley et al. 2007; Baarendse and Vanderschuren 2012; Baarendse et al. 2013b; Sanchez-Roige et al. 2014a, b, 2016). Indeed, previous studies have shown that after acute or chronic treatment with alcohol, increases in impulsive action are only observed during challenges with long or variable ITIs (Peña-Oliver et al. 2009; Walker et al. 2011; Irimia et al. 2015; Sanchez-Roige et al. 2016, but see Peña-Oliver et al. 2015). That said, in the present study, the increase in premature responses in HD was modest, as it only occurred during the first session in which the ITI was suddenly prolonged. This observation is comparable with a human study in which an acute alcohol challenge provided to young adults with a family history of AUD resulted in an increase in premature responding only during the first and not a second challenge session (Sanchez-Roige et al. 2016). Taken together, our findings therefore show that whereas HD display behavioral characteristics of AUD (Spoelder et al. 2015a), this is not necessarily paralleled by impaired decision making but might be more related to increased impulsive action.

We observed less impulsive choice behavior in HD in the DRT. That is, the HD showed a higher preference for the delayed reward during the training phases of the DRT, in which the delay to the large reward was relatively short. Moreover, the principal component analysis for the second experiment revealed that alcohol intake and DRT performance loaded onto the same factor. The difference in impulsive choice between groups was no longer apparent when the final version of the DRT was implemented, with a maximum delay to the large reward of 60 s. When the animals were then tested in a DRT version with decreasing, instead of increasing delays within the session, both LD and HD adapted choice behavior in a comparable manner. Hence, it is not likely that the increase in choice for the large reward during DRT training reflects perseverative responding in HD. Alternatively, we reasoned that the transient reduction in impulsive choice is related to the time period between IAA and the DRT test phases, i.e., reduced impulsive choice is only apparent during early withdrawal from excessive alcohol intake. To test this possibility, the animals were re-exposed to alcohol for six 24 h IAA sessions. Importantly, the alcohol intake and alcohol preference of the LD and HD during these re-exposure sessions did not differ from the intake and preference during previous sessions with 24 h IAA, again demonstrating that the difference in alcohol-directed behavior between LD and HD is a consistent trait. This re-exposure to IAA resulted in a re-emergence of the differences between HD and the LD and WAT. However, this effect was of a markedly lesser magnitude than the reduction in impulsive choice during DRT training. Because the level of alcohol intake in the re-exposure sessions was similar to previous alcohol consumption sessions, altered alcohol consumption cannot explain these findings. Interestingly, post hoc analyses indicated a decrease in the percentage choice for the large delayed reward for the LD and WAT but not for the HD. These findings suggest that either after a break of testing or after additional test sessions, an increase in impulsive choice occurs, which may be inhibited by intake of a substantial amount of alcohol. Another possibility is that these differences in impulsive choice are only observed when relatively short delays to the large reward are used. We observed that the variability in choice behavior between rats declined as the delays were increased to a final 60 s, which has been observed by others as well (Flagel et al. 2010). It has been reported that alcohol-naïve alcohol-preferring rats and mice show enhanced impulsive choice behavior in the DRT (Wilhelm and Mitchell 2008; Oberlin and Grahame 2009; Beckwith and Czachowski 2014; Perkel et al. 2015), although this is not a general finding (Wilhelm et al. 2007; Wilhelm and Mitchell 2012). Importantly, the delays used in these studies (8, 16, and 25 s) are in the range of the delays we used during the early phases of the DRT in the present study (12, 24, and 48 s). Thus, our findings indicate that HD display lower impulsive choice behavior, but this is only apparent under relatively unchallenging task conditions (i.e., short delays), and that this can be mitigated by prolonged abstinence from alcohol.

Acute alcohol treatment improved decision making in the rGT in LD and HD. This observation is in contrast to previous findings, showing impaired or unaltered decision making upon acute alcohol exposure in humans and rodents (Lane et al. 2004; George et al. 2005; Ramaekers and Kuypers 2006; Mitchell et al. 2011; Peña-Oliver et al. 2014; Spoelder et al. 2015b). Alcohol-induced perseverance in responding may have increased the percentage choice for the well-established preferred option in this study, although it remains unclear why this occurred after IAA and not in alcohol-naïve rats or in rats that were passively pre-exposed to alcohol (Spoelder et al. 2015b). Acute alcohol exposure increased impulsive choice by increasing the preference for the small immediate reward in all three subgroups, which is in line with previous studies (Poulos et al. 1995; Tomie et al. 1998; Evenden and Ryan 1999; Olmstead et al. 2006; Wilhelm and Mitchell 2012). These findings are consistent with the increases in impulsive choice after acute alcohol exposure in heavy alcohol drinking individuals compared to light drinkers (Marczinski et al. 2007; King et al. 2011; Reed et al. 2012, but see Sanchez-Roige et al. 2016).

The better performance of HD during nose-poke training, DRT training, and the rGT, lead us to think that HD might attribute more value to primary or conditioned rewards. It has been proposed that poorly controlled alcohol drinking may be due to an exaggerated sign-tracking conditioned response, resulting in increased consumption of alcohol when confronted with alcohol-related cues (Olmstead et al. 2006; Tomie and Sharma 2013). Indeed, studies in humans have reported an association between an approach tendency towards reward-predictive cues and individual levels of alcohol consumption (Field and Cox 2008; Stacy and Wiers 2010). In the present study, we observed enhanced approach towards a reward-predictive cue in HD compared to LD in experiment 1. Moreover, in the principal component analysis of this experiment, alcohol intake and response bias (i.e., an index of sign- vs. goal-tracking) in the Pavlovian-conditioned approach task loaded onto the same factor. In the second experiment, we found that LD, HD, and WAT did not differ in conditioned approach prior to IAA. Rather, the LD and HD showed a tendency towards goal-tracking behavior. This is somewhat consistent with a recent study, which reported goal-tracking in alcohol-naïve rats, which was more pronounced in alcohol-preferring rats (Peña-Oliver et al. 2015). After IAA, all rats were tested again in the Pavlovian-conditioned approach task, in which they now showed increased sign-tracking behavior. The principal component analysis of the rats in experiment 2 showed that the response bias score before IAA loaded onto the same factor as the response bias score after IAA access. These observations extend previous work that reported increases in sign-tracking behavior after a period of alcohol exposure (McClory and Spear 2014; Spoelder et al. 2015c). The absence of a group difference in approach behavior after IAA in experiment 2 may be related to the fact that these rats had already been tested in the Pavlovian-conditioned approach task prior to IAA. In other words, a history of high alcohol intake alters the acquisition of Pavlovian-conditioned approach, so that HD rats are more biased to approach the reward-associated cue. However, with extended testing in a Pavlovian-conditioned approach task, sign-tracking may become the predominant behavior (Clark et al. 2013), so that LD rats come to display a sign-tracking phenotype as well, masking differences between LD and HD rats. Alternatively, the differences in Pavlovian-conditioned approach between the groups could be due to an inherent predisposition of the animals to sign- or goal-track. For example, it has been demonstrated that the distribution of sign- and goal-trackers can vary from batch to batch and from vendor to vendor (Fitzpatrick et al. 2013). However, we do not think that this can explain the present data. Although the WAT group in experiment 2 seemed to show more sign-tracking behavior compared with LD and HD, no difference between the LD and HD groups was apparent in experiment 2. In addition, in experiment 2, Pavlovian-conditioned approach behavior in the three groups of rats developed in a comparable fashion. Taken together, these results show that HD attribute more value to reward-associated cues. This enhanced sign-tracking response in HD is not a pre-existing trait, but more likely to be the consequence of a high level of alcohol intake, and the alcohol-induced increase in sign-tracking can be masked by prolonged training.

The increase in sign-tracking, impulsive action and preference for a large delayed reward in HD is consistent with previous studies that showed that sign-trackers display reduced impulsive choice, but enhanced impulsive action (Flagel et al. 2010; Lovic et al. 2011). With regard to the relationship between impulsivity and alcohol use, the present data suggest that high alcohol intake is associated with both impulsive action and impulsive choice, but in opposite directions. Interestingly, in a previous study in humans, it was shown that the relationship between automatic alcohol approach tendencies and alcohol consumption was not dependent on the level of impulsivity, as measured by the Barratt Impulsiveness Scale, the DRT, and a Go/No-Go Task, indicating that the multiple components of impulsivity and the automatic approach tendencies each explain a unique variance in alcohol consumption (Christiansen et al. 2012).

To conclude, the present results show a relationship between voluntary alcohol consumption and decision making, impulsivity and Pavlovian-conditioned approach. HD perform better than LD in both the rGT and DRT, allowing them to maximize their gains. In addition, HD show increased approach towards a food-predicting cue, which was the result of alcohol intake rather than a pre-existing trait. Together, these findings provide novel insight into the underlying mechanisms for individual differences in alcohol consumption that is propelled by more efficient reward- and cue-driven behavior.
